# Machine Learning–Guided Fluid Resuscitation for Acute Pancreatitis Improves Outcomes

**DOI:** 10.14309/ctg.0000000000000825

**Published:** 2025-01-24

**Authors:** Niwen Kong, Patrick Chang, Ira A. Shulman, Ubayd Haq, Maziar Amini, Denis Nguyen, Farhaad Khan, Rachan Narala, Nisha Sharma, Daniel Wang, Tiana Thompson, Jonathan Sadik, Cameron Breze, David C. Whitcomb, James L. Buxbaum

**Affiliations:** 1 Division of Gastroenterology, Department of Medicine, University of Southern California, Los Angeles, California, USA;; 2 Department of Pathology, University of Southern California, Los Angeles, California, USA;; 3 Ariel Precision Medicine, Pittsburgh, Pennsylvania, USA;; 4 Division of Gastroenterology, Department of Medicine, University of Pittsburgh, Pittsburgh, Pennsylvania, USA.

**Keywords:** pancreatitis, artificial intelligence, machine learning, resuscitation, systemic inflammatory response syndrome, respiratory insufficiency

## Abstract

**INTRODUCTION:**

Ariel Dynamic Acute Pancreatitis Tracker (ADAPT) is an artificial intelligence tool using mathematical algorithms to predict severity and manage fluid resuscitation needs based on the physiologic parameters of individual patients. Our aim was to assess whether adherence to ADAPT fluid recommendations vs standard management impacted clinical outcomes in a large prospective cohort.

**METHODS:**

We analyzed patients consecutively admitted to the Los Angeles General Medical Center between June 2015 and November 2022 whose course was richly characterized by capturing more than 100 clinical variables. We inputted these data into the ADAPT system to generate resuscitation fluid recommendations and compared with the actual fluid resuscitation within the first 24 hours from presentation. The primary outcome was the difference in organ failure in those who were over-resuscitated (>500 mL) vs adequately resuscitated (within 500 mL) with respect to the ADAPT fluid recommendation. Additional outcomes included intensive care unit admission, systemic inflammatory response syndrome (SIRS) at 48 hours, local complications, and pancreatitis severity.

**RESULTS:**

Among the 1,083 patients evaluated using ADAPT, 700 were over-resuscitated, 196 were adequately resuscitated, and 187 were under-resuscitated. Adjusting for pancreatitis etiology, gender, and SIRS at admission, over-resuscitation was associated with increased respiratory failure (odd ratio [OR] 2.73, 95% confidence interval [CI] 1.06–7.03) as well as intensive care unit admission (OR 2.40, 1.41–4.11), more than 48 hours of hospital length of stay (OR 1.87, 95% CI 1.19–2.94), SIRS at 48 hours (OR 1.73, 95% CI 1.08–2.77), and local pancreatitis complications (OR 2.93, 95% CI 1.23–6.96).

**DISCUSSION:**

Adherence to ADAPT fluid recommendations reduces respiratory failure and other adverse outcomes compared with conventional fluid resuscitation strategies for acute pancreatitis. This validation study demonstrates the potential role of dynamic machine learning tools in acute pancreatitis management.

## Graphical abstract

**Figure:**
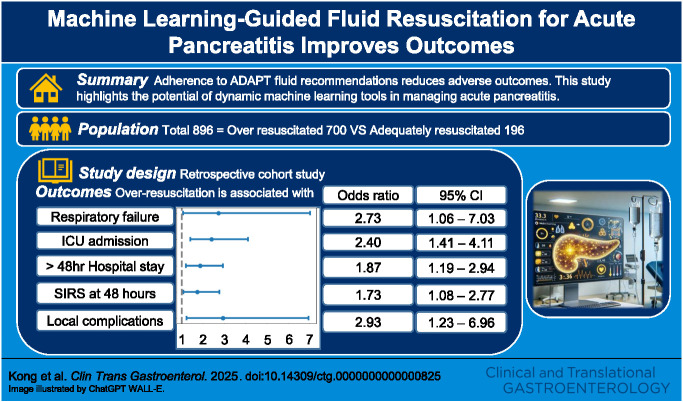

## INTRODUCTION

Acute pancreatitis (AP) is a complex management challenge because of its multifaceted etiology, variable clinical course, and potential for severe complications. The intricate interplay of local and systemic factors often makes the trajectory of disease difficult to predict (1,2), thus making it challenging to optimize the treatment strategies. One crucial aspect of AP management is fluid resuscitation, which aims to prevent hypovolemia to maintain organ perfusion and reduce complications (3,4). However, over-resuscitation has also been associated with respiratory complications and abdominal compartment syndrome (5,6). Recently, the Early Weight-Based Aggressive vs ModeraTE Goal-DiRected Fluid ResuscitAtion (WATERFALL) multicenter, randomized controlled trial has shown that aggressive hydration in unselected patients did not reduce moderate/severe pancreatitis but instead resulted in increased fluid overload and prolonged length of hospital stay (7). Systematic review and meta-analysis suggests that ample fluids may be helpful in mild pancreatitis, but aggressive resuscitation in the later course of more severe pancreatitis might be associated with higher mortality, sepsis, and compartment syndrome (8). However, among existing studies, the initial volume and rate of fluid administered varies substantially as well as the total fluid administrated (8). The optimal amount of fluid to administer in AP remains an unresolved challenge in clinical practice. Real-time fluid resuscitation guidance, tailored to the unique characteristics of each patient, is needed.

With the advancement in artificial intelligence (AI) and machine learning (ML), various computer-aided clinical decision tools have been developed to predict disease trajectory with increasing sophistication. Artificial neural networks were first used to predict the development of organ failure, prolonged hospital stay, and mortality in AP (9). Prediction of AP disease course has been further enhanced using cutting-edge AI tools including eXtreme Gradient Boosting tree and leaf decision enhanced technology (10,11).

The novel AP severity and trajectory modeling tool, Ariel Dynamic Acute Pancreatitis Tracker (ADAPT), has been validated for predicting severity using the University of Pittsburgh Medical Center pilot and the international Acute Pancreatitis Patient Registry to Examine Novel Therapies in Clinical Experience cohort (12). In addition to severity prediction, ADAPT is a clinical decision support system (CDS) built to guide AP treatment including fluid volume resuscitation strategy based on the individual patient's immediate biochemical and clinical status. Nevertheless, its utility has yet to be tested in a real-world setting. The aim of our study is to explore whether adherence to fluid recommendations by ADAPT is associated with differences in clinical outcomes.

## METHODS

### ADAPT tool

**Figure 1.**
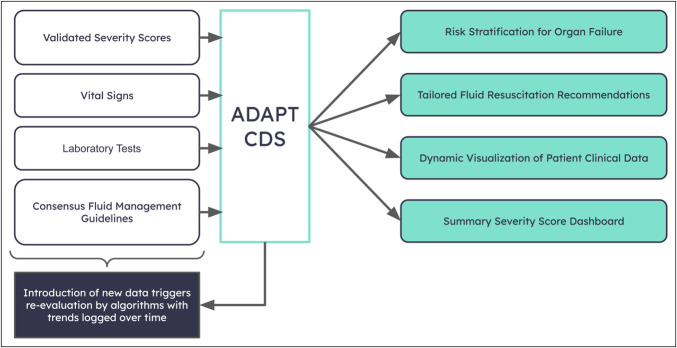
ADAPT CDS uses real-time input of individual patient physiologic data and consideration of consensus guidelines and scoring systems to make personalized fluid resuscitation recommendations as well as predict severity and organ failure. Ariel algorithm to predict fluid needs during acute pancreatitis. ADAPT, Ariel Dynamic Acute Pancreatitis Tracker; CDS, clinical decision support system.

The ADAPT (Patent 146945.00101 US Publication No. 20200176119A1 dated 6.4.20) is a CDS created to assist healthcare professionals in managing individual patients with AP (Figure 1). It uses a set of mathematical and rule-based models to simulate characteristics of individual subjects. These models: (i) adjust for factors such as age, sex, and body composition (e.g., fat) to determine the size of various compartments in an individual case; (ii) use biophysical trajectory models to monitor the dynamic phases of the disease in various organ systems; and (iii) implement predictive models to establish links between these trajectories and potential outcomes, both with and without medical interventions (12). Thus, unlike a higher-order AI application, ADAPT's equation-based modeling approach generates exact results that are fully trackable and reproducible (i.e., no “black box” functions).

### Population

All patients presenting with AP to the Los Angeles County General Medical Center (LAGMC) between June 2015 and November 2022 were captured in the LAGMC prospective cohort. Institutional Review Board approval from the University of Southern California Health Sciences Institutional Review Board was obtained before its initiation. Patients with AP were identified through a real-time emergency department patient tracking system and laboratory alerts of abnormal pancreatic enzymes. Patients were then further evaluated for diagnosis of AP if they met 2 of 3 criteria: lipase greater than 3 times the upper limit of normal; characteristic abdominal pain; or imaging consistent with AP.

For each patient in the cohort, more than 150 clinically relevant variables were recorded over the course of the admission including but not limited to demographics, pancreatitis etiology, comorbidities, substance use history, vital signs, visual analog pain score, inpatient pain medications, total intravenous fluid given per 24-hour period, biochemical parameters, imaging results, intensive care unit (ICU) admission, and length of stay (LOS). The presence of systemic inflammatory response syndrome (SIRS) was defined as 2 of 4 criteria: heart rate >90 beats per minute; respiration >20 per minute; temperature <36 or >38°C; or white blood cell <4,000 or >12,000/mm^3^ (13), ICU admission, and hospital LOS.

### Use of ADAPT to predict fluid requirements

Parameters contained within the database, including age, gender, height, weight, admission vitals (temperature, blood pressure, heart rate, respiratory rate, oxygen saturation, and amount of supplemental oxygen given), and white blood cells were entered into ADAPT to generate initial (within first 24 hours) fluid recommendations for the patients in the Los Angeles General Hospital (LAG) cohort. Patients were excluded if they were transferred from or out to another hospital, left against medical advice, presented with clinical or radiographic evidence of chronic pancreatitis or cirrhosis, had stage IV or stage V chronic kidney disease, or New York Heart Association (NYHA) III or IV heart failure.

The specific ADAPT recommendations for each patient were compared with the actual fluid administered in the first 24 hours as recorded for the cohort. The adequately resuscitated group was defined as patients whose first 24-hour fluid resuscitation was within ±500 mL from ADAPT recommendation. The over-resuscitated group was defined as the first 24-hour fluid resuscitation of more than 500 mL over ADAPT recommendation. The under-resuscitated group received at least 500 mL less than the recommended fluid during the first 24 hours.

### Outcomes

The aim of the analyses was to compare the outcomes for those who were over-resuscitated vs adequately resuscitated. The primary outcome was defined as the development of new-onset pulmonary, cardiac, or renal failure. Modified Marshall organ failure score ≥2 was used to identify presence of organ failure (14). Additional cardinal outcomes included the development of local complications (i.e., pseudocyst and necrosis) or moderately severe and severe pancreatitis both as defined by the revised Atlanta classification (15). Furthermore, ICU admission, ICU and hospital LOS, and SIRS at 48 hours were compared. Additional outcomes of interest included 24- and 48-hour morphine equivalent administration, new pleural effusions, and biochemical parameters including 48-hour hematocrit and calcium levels.

In addition, we aimed to explore the ability of ADAPT to guide fluid therapy in patients with more predicted severe disease by performing a comparison of over vs adequate resuscitation in patients with a Harmless Acute Pancreatitis Score (HAPS) >1. We also compared the population and outcomes of those who were under-resuscitated, defined as fluids <500 mL of ADAPT, vs adequately resuscitated.

### Analysis

Univariate analysis was first performed of all baseline parameters to identify potential confounders in the planned comparison of adequate resuscitation vs over-resuscitation. This was performed using logistic regression for categorical variables and linear regression or the Mann-Whitney test for continuous variables depending on normality of the distribution. After assessment for collinearity, potentially influential variables were included in the multivariate logistic and linear regression models used to compare the clinical outcomes in the 2 groups. The presence of SIRS at admission as well as the SIRS score, which is defined as the number of criteria (13,16–18), correlates with severe disease course (13,16). Therefore, all analyses were controlled for baseline (admission) SIRS to adjust for severity.

### Sensitivity analyses

To further control for pancreatitis severity and baseline medical problems, we adjusted all multivariate analyses for Glasgow Score, Glasgow score >3, Panc3 score, HAPS, Bedside Index of Severity in Acute Pancreatitis (BISAP) score, BISAP score >3, and Charlson comorbidity index. We also presented these elements as part of the baseline comparison for patients who were adequately resuscitated and over-resuscitated. In addition, to account for the presence of baseline local complications (e.g., baseline necrosis, organized fluid collection, and pseudocyst), we controlled these analyses for the baseline presence of the finding.

To define the impact in more severe diseases, we performed a subanalysis comparing the main outcomes in those over-resuscitated vs adequately resuscitated in patients with HAPS ≥1.

We used the same analysis strategy to compare the populations and clinical outcomes of those who were under-resuscitated vs adequately resuscitated according to ADAPT recommendations.

All analyses were performed using STATA version 16.0 (College Station, TX).

## RESULTS

### Population

Between June 2015 and November 2022, 1,409 unique patients with AP were admitted to LAGMC, and their detailed clinical profile was captured in the prospective database (see Supplementary Figure 1, Supplementary Digital Content 1, http://links.lww.com/CTG/B282). We excluded patients in a stepwise fashion because some of them met multiple exclusion criteria. Initially, 70 patients with a history of chronic pancreatitis were excluded, followed by 75 patients with computed tomography or ultrasound findings showing signs of cirrhosis within 6 months from admission. Subsequently, 85 patients with a history of stage IV or stage V chronic kidney disease were excluded, along with an additional 18 patients with NYHA III or IV heart failure. Finally, we excluded 24 patients who were transferred to an outside hospital or left against medical advice, as well as 54 patients with missing data.

After entry of the core clinical and laboratory parameters into the ADAPT system in comparison with the fluids administered, we found that 700 were over-resuscitated, 196 were adequately resuscitated, and 187 were under-resuscitated (Table 1). The mean fluid administered in the adequately resuscitated group was 2,465.4 ± 539.3 mL, which accorded with the ADAPT fluid recommendation of 2,435 ± 417 mL (Table 2). Administered fluid in the over-resuscitated group, 4,876.9 ± 1,661.6 mL, far exceeded ADAPT fluid recommendations, 2,372 ± 436 mL (*P* < 0.001).

**Table 1.** Baseline features population

	Adequately resuscitated		Over-resuscitated		*P* value
	Mean	SD	Mean	SD	
ADAPT recommended fluid first 24 hr (mL)	2,435.1	417.4	2,372.0	436.3	0.07
Actual fluid administered first 24 hr (mL)	2,465.4	539.3	4,876.9	1,661.6	0.00
Mean difference ADAPT vs actual fluid	30.3	294.1	2,506.6	1,583.4	0.00
Age (yr)	45	16	43	15	0.04
BMI (kg/m^2^)	31.72	12.8	28.9	7.2	0.00
BUN (mg/dL)	13.4	6.3	14.0	8.0	0.96
Creatinine (mg/dL)	0.75	0.22	0.80	0.34	0.06
GFR (mL/min)	105	23	105	25	0.90
Calcium (mg/dL)	9.2	0.8	9.2	0.8	0.92
Lipase (U/L)	2,076	2,081	2,291	2,176	0.22
Total cholesterol (mg/dL)	221	182	218	155	0.91
Pain score (visual analog)	7.4	3.1	7.5	3.1	0.54
	N	%	N	%	*P* value
Male	89	45.4	376	53.7	0.04
Female	107	54.6	324	46.3	
Race					
Hispanic	169	86.2	591	84.4	0.87
White	6	3.1	25	3.6	
Black	8	4.1	29	4.1	
Asian	6	3.1	33	4.7	
Other	7	3.6	22	3.1	
Current smoker	27	13.9	104	14.9	0.73
Heavy EtOH	36	18.5	188	27.0	0.02
Familial history of pancreatic cancer	1	0.5	4	0.6	0.92
Etiology					
EtOH	26	13.8	166	24.2	0.00
Gallstone	112	59.3	325	47.4	
Other	51	27.0	195	28.4	
SIRS at admission	38	19.4	177	25.3	0.09
Admission Glasgow score					
0	88	44.9	291	41.6	0.44
1	63	32.1	245	35.0	
2	35	17.9	112	16.0	
3	10	5.1	42	6.0	
≥4	0	0.0	10	1.4	
HAPS positive	81	41.3	349	49.9	0.04
BISAP severe	2	1.0	21	3.0	0.12
Charlson comorbidity score					
0	99	50.5	374	53.4	0.15
1	37	18.9	166	23.7	
2	31	15.8	85	12.1	
3	12	6.1	42	6.0	
4	9	4.6	19	2.7	
≥5	8	4.1	14	2.0	
DM					
Type 1	6	3.1	21	3.0	0.31
Type 2	45	23.0	150	21.4	
Insulin	15	8.0	58	8.5	0.82
HTN	45	23.3	165	24.3	0.77
HLD	23	11.9	87	12.9	0.73
Statin	16	8.3	62	9.2	0.72
Fibrate	5	2.6	16	2.4	0.85
CKD					
1	149	76.0	530	75.8	0.26
2	39	19.9	117	16.7	
3	8	4.1	49	7.0	

ADAPT, Ariel Dynamic Acute Pancreatitis Tracker; BISAP, Bedside Index of Severity in Acute Pancreatitis; BMI, body mass index; BUN, blood urea nitrogen; CKD, chronic kidney disease; DM, diabetes mellitus; EtOH, ethanol; GFR, glomerular filtration rate; HAPS, Harmless Acute Pancreatitis Score; HLD, hyperlipidemia; HTN, hypertension; SIRS, systemic inflammatory response syndrome.

**Table 2.** Clinical outcomes

Outcomes	Adequately resuscitated N (%)	Over-resuscitated N (%)	Odds ratio^a^	95% CI
New respiratory failure	2 (1.0)	56 (8.0)	2.73	1.06 to 7.03
New circulatory failure	2 (1.0)	14 (2.0)	0.97	0.20 to 4.64
New renal failure	5 (2.6)	15 (2.1)	0.51	0.13 to 2.05
Local pancreatitis complications	9 (5.8)	92 (17.0)	2.93	1.23 to 6.96
Pancreatic pseudocyst	2 (1.3)	22 (4.1)	2.30	0.52 to 10.12
Necrosis	4 (2.6)	45 (8.3)	2.75	0.95 to 7.95
Organized fluid collection	3 (1.9)	25 (4.6)	2.11	0.62 to 7.19
New pleural effusion	4 (2.0)	42 (6.0)	2.76	0.97 to 7.86
Moderately severe pancreatitis	17 (8.7)	95 (13.6)	1.52	0.92 to 2.71
Severe pancreatitis	4 (2.0)	42 (6.0)	2.44	0.85 to 7.01
Required ICU admission	19 (9.7)	155 (22.1)	2.40	1.41 to 4.11
ICU length of stay >48 hr	12 (6.1)	88 (12.6)	1.63	0.93 to 2.87
Hospital length of stay >48 hr	156 (79.6)	594 (84.9)	1.87	1.19 to 2.94
SIRS at 48 hr after admission	27 (13.8)	159 (22.7)	1.73	1.08 to 2.77
Death	2 (1.0)	7 (1.0)	0.71	0.14 to 3.56
Readmission	20 (10.2)	84 (12.0)	1.16	0.68 to 1.98
	Adequately resuscitated Mean (SD)	Over-resuscitated Mean (SD)	Coefficient^a^	95% CI
Length of stay	6 (4)	7 (10)	1.08	−0.33 to 2.48
BUN max during hospitalization	14 (7)	15 (11)	1.10	−0.53 to 2.72
First 24-hr total oral morphine equivalent	27.3 (35.8)	37.9 (42.8)	9.42	2.70 to 16.15
First 48-hr total oral morphine equivalent	19.4 (37.5)	27.2 (46.5)	6.39	−0.94 to 13.72

BUN, blood urea nitrogen; CI, confidence interval; ICU, intensive care unit; SIRS, systemic inflammatory response syndrome.

a All analyses are multivariate controlling for gender, pancreatitis etiology, and baseline SIRS.

Comparison of the over-resuscitated vs adequately resuscitated group indicated that the former included more men and patients whose etiology was alcoholic pancreatitis (Table 1). There was a trend toward more frequent SIRS at admission in the over-resuscitated group, but this did not reach statistical significance (*P* = 0.09). Although the baseline HAPS was higher in the over-resuscitated group, there was no difference in admission Glasgow or BISAP scores. There was no difference in tobacco use, race or ethnicity, or use of statins between those who were overly or adequately resuscitated. There was also no difference in baseline Charlson comorbidity index or the proportion with concomitant diabetes or chronic kidney disease between the 2 groups (Table 1). There was also no difference in the admission pain score, blood urea nitrogen, calcium, or albumin.

### Outcomes

#### Organ failure and local complications

After adjusting for gender, pancreatitis etiology, and SIRS at admission (as a metric of severity), over-resuscitation was associated with significant higher risk of respiratory failure (2.73, 95% confidence interval [CI] 1.06–7.03) but no difference in circulatory or renal failure (Table 2). There was no difference in moderately severe/severe and severe pancreatitis between the 2 groups. Overall, patients who were over-resuscitated were more likely to develop local pancreatitis complications (2.93, 95% CI 1.23–6.96).

#### Additional clinical outcomes

In multivariate analysis, over-resuscitation was associated with more SIRS 48 hours after admission (odd ratio [OR] 1.73, 95% CI 1.08–2.77), required ICU admission (2.40, 95% CI 1.41–4.11), and necessitated ≥48 hours of hospital LOS (1.87, 95% CI 1.19–2.94) (Table 2).

Notably, the over-resuscitated group required significantly more total oral morphine equivalent (coefficient 9.42, 95% CI 2.70–16.15) within 24 hours of admission. There was no difference in mortality or readmission.

### Sensitivity analyses

Multivariate analyses for primary and secondary outcomes were adjusted using a variety of severity scores including Glasgow, HAPS, Panc3, and BISAP, and our findings of greater new onset respiratory failure, local complications, and ICU admission with over-resuscitation remained robust (see Supplementary Table 1, Supplementary Digital Content 2, http://links.lww.com/CTG/B283). In addition, adjustment for the baseline comorbidity index did not materially impact our clinical outcomes (see Supplementary Table 1, Supplementary Digital Content 2, http://links.lww.com/CTG/B283). In a sub-analysis of patients with an elevated HAPS, the magnitude of the protective effect and other beneficial outcomes was greater; for example, OR respiratory failure in over-resuscitated vs adequately resuscitated was 9.9 (95% CI 1.3–74.3) (see Supplementary Table 2, Supplementary Digital Content 2, http://links.lww.com/CTG/B283).

Among the 9 patients who were adequately resuscitated and developed a local complication, 4 were present at admission. Of the 92 patients who were over-resuscitated with a local complication, 52 were present at baseline. Over-resuscitation remained a significant predictor of overall local complication (OR 7.4, 95% CI 1.3–42.7) after including baseline local complication as a cofactor in the multivariate analysis. Although there were trends toward increased specific local complications, pancreatic pseudocyst (OR 3.7, 95% CI 0.3–45.1), necrosis (OR 1.7, 95% CI 0.4–7.6), and organized fluid collections (OR 8.1, 95% CI 0.8–86.4), these are still statistically insignificant after adjusting for their baseline presence.

### Under-resuscitated patients

In the prospective cohort, 187 patients received more than 500-mL less fluid than recommended by the ADAPT system. Although they had a similar age, body mass index, and pancreatitis etiology, they had a lower mean creatinine (see Supplementary Table 3, Supplementary Digital Content 2, http://links.lww.com/CTG/B283). They also were less likely to have baseline SIRS, 10.7% vs 19.4%. There was no difference in the clinical outcomes of organ failure, ICU admission, LOS, or moderately severe/severe pancreatitis, although there was a lower likelihood of SIRS at 48 hours, 8% vs 13.8% (see Supplementary Table 4, Supplementary Digital Content 2, http://links.lww.com/CTG/B283).

## DISCUSSION

AP remains a challenge given its unpredictable course and need for highly individualized management. Extensive systems to forecast its clinical course have fallen short, and a uniform fluid management strategy is lacking. In the context of the ongoing AI revolution, mathematical models to simultaneously process extensive data are being used to synthesize information and propose the next steps in medicine and society in general. In this study, we test the ability of a dynamic ML system designed to analyze the course of pancreatitis course, ADAPT, to guide the fluid therapy of a large diverse cohort of patients.

Recently, randomized controlled trials have demonstrated that AI improves such key parameters as the adenoma detection rate, number of adenomas detected per colonoscopy, and the adenoma miss rate (17–19). Cholangioscopy enhanced using ML technology performs better than tissue sampling for the classification of malignant bile duct strictures (20).

Thus far, applications of AI and ML in AP have been to address the longstanding issue of severity prediction. Since the mid-1970s, extensive tools have been developed or used to predict the development of severe pancreatitis including Ranson's and Glasgow, the Acute Physiology and Chronic Health Evaluation, BISAP, and the HAPS to name a few (21–25). Although high scores are specific for severe pancreatitis development, none of the routinely used scoring system are sensitive. Consequently, their positive predictive value and positive likelihood ratios for severe pancreatitis only approach the modest level of 50% (26). By applying a high-complexity combination of 12 different scoring systems in 12 different ways, Mounzer et al improved the accuracy to 84% but was judged by the authors to be too cumbersome for clinical use (27).

The ADAPT uses real-time data and uses machine learning technology not only to generate the results for major scoring systems (i.e., HAPS and BISAP score) but to also apply the complex Mounzer rules to predict pancreatitis severity (12). Testing in 2 major prospective cohorts reveals high accuracy of this CDS tool for AP severity prediction. Recently, investigators have used sophisticated ML-based strategies including eXtreme Gradient Boosting tree and leaf decision and support vector machine technology to develop models for severity prediction and defined impact of individual factors identified by AI using Shapely Additive exPlanations plots (10,11).

The ADAPT model takes it a step further by making treatment recommendations in addition to predicting severity. The system inputs real-time patient variables into mathematical and rule-based models of physiology to define fluid needs (12). The comparison of volume recommendations made by ADAPT in comparison with the clinical judgment of the physicians at our center was the focus of this study. In this study, we found that patients whose fluid administration accorded ADAPT recommendations in the first 24 hours had a better clinic course than those who did not (3,4). After adjusting for pancreatitis severity, patients whose initial 24-hour fluid resuscitation volume was within 500 mL of the ADAPT recommendation were nearly 3-fold less likely to develop respiratory failure and were substantially less likely to require ICU admission, develop local complications, and have a hospital stay >48 hours.

Interestingly, most patients were over-resuscitated relative to ADAPT recommendations. We theorize that this represents the influence of 2 decades of guidance favoring indiscriminate aggressive initial resuscitation of AP as codified in several clinical practice guidelines (28,29). Our findings, that a more measured approach improves outcomes, accord with the principal results of the recent WATERFALL trial (7). Our findings of over-resuscitation and adverse outcomes agree with emerging evidence about the physiology of AP resuscitation. Although aggressive hydration in the first phase of pancreatitis prevents renal dysfunction and hypovolemia, excessive fluid administration later in the disease course exacerbates third spacing and overwhelms systems to return fluid to the intravascular system (30–32). This may result in peripheral edema, rhabdomyolysis, and ascites but of even greater importance pulmonary edema, effusion, and respiratory failure. We found that the later problem was more frequent in those whose resuscitation exceeded ADAPT guidelines, and on average, this was by more than 2 L during the first 24 hours of management. Detailed trajectory modeling of ADAPT may help to optimize the competing goals of restoration of intravascular fluids vs pulmonary complications of resuscitation.

Our study has several limitations. First, we used an existing database of AP patients. Nevertheless, our cohort has been prospectively developed and is richly characterized with >100 variables including all inputs needed for the ADAPT AI system and relevant outcome metrics. An advantage is that we were able to compare the fluid administered in real-world practice vs ADAPT recommendations on a patient-by-patient basis; this functionally generated a repeated-measures clinical trial of >800 subjects. Another consideration is that there were potential underlying differences in those whose fluid management was consistent with vs much greater than ADAPT recommendation. Indeed, the HAPS was higher in this group. Nevertheless, the presence of baseline SIRS as well as Glasgow, BISAP, and Charlson comorbidity scores of the 2 groups was not significantly different. In addition, multivariate analyses adjusting for differences in SIRS, BISAP, Glasgow, HAPS1, and Charlson comorbidity indicated that our primary findings were robust (16).

Fluid management in those with moderately severe and severe pancreatitis is the most challenging and uncertain. In the WATERFALL trial and its predecessor trial, patients with clinical signs of shock, respiratory failure, or renal failure were excluded (7,33). Systematic reviews have shown only limited studies of fluid management in these severe pancreatitis have compared aggressive (5 mL/kg/hr) to extremely aggressive (10 mL/kg/hr) fluid strategies that are rarely used in clinical practice (34–37). We are optimistic that ADAPT may help to guide fluid management in more severe pancreatitis, given that the magnitude of the protective effect of following ADAPT guidance was greater in the subset of patients with predicted moderately severe pancreatitis; the OR of respiratory failure in those over-resuscitated vs adequately resuscitated was 9.9 in those with HAPS >1 vs 2.7 for the entire group.

Although our primary focus was to compare adequately resuscitated vs over-resuscitated patients, during the study period, 187 received less than recommended by ADAPT. This group had a lower creatinine and less SIRS at baseline, potentially suggesting more milder disease or less need for resuscitation. There were no substantive differences in clinical outcomes with the exception of less persistent SIRS in those who were under-resuscitated. This interesting finding accords with a strategy of more targeted and measured resuscitation in AP.

It also further underscores the immediate need for a randomized controlled trial of ADAPT vs weight-based algorithms and clinical algorithms is the next to further define the role of AI in AP. In addition, there is a need to explain why a more measured approach to fluid treatment reduced local complications in our study. We anticipate that this will require a translational component to define. In theory, fluid overload may exacerbate systemic inflammatory processes, which may feedback to events in the peripancreatic milieu. This may be exacerbated in patients with underlying metabolic syndrome, which may impact fluid exchange between adipose and other body tissue.

In summary, our findings support the use of computerized ML tools to enhance patient care and guide personalized treatment strategies in the management of AP. Using a rich data set of pancreatitis, this study suggests that the use of an ADAPT-guided fluid resuscitation strategy during the first 24 hours improves overall outcomes driven by a reduction in the critical outcome of respiratory failure. The ability to simultaneously integrate multiple aspects of an individual's minute-to-minute physiology by powerful and dynamic ML systems may improve other aspects of the treatment of this complex disease. This study underscores the potential for AI to direct patient care in the field of pancreatology.

## CONFLICTS OF INTEREST

**Guarantor of the article:** James L. Buxbaum, MD.

**Specific author contributions:** N.K., P.C., J.S., D.C.W., and J.L.B.: concept and design. N.K., I.A.S., U.H., M.A., D.N., F.K., R.N., N.S., D.C.W., T.T., and J.L.B.: acquisition of data. N.K., P.C., D.C.W., and J.L.B.: statistical analysis and interpretation of data. N.K., P.C., I.A.S., C.B., D.C.W., and J.L.B.: drafting and revision of the manuscript.

**Financial support:** None to report.

**Potential competing interests:** C.B. is an employee of Ariel. D.C.W. is the Chief Scientific Advisor of Ariel. Ariel is the developer and owner of the Ariel Dynamic Acute Pancreatitis Tracker (ADAPT).

**Previous presentation:** The study was presented in abstract format at the 2023 and 2024 meetings of the American Gastroenterology Association.

**Study Highlights**

**WHAT IS KNOWN**

✓ Artificial intelligence and machine learning are transforming many aspects of medical practice including the diagnosis of gastrointestinal lesions.
✓ The role of machine learning in pancreatology has thus far been used to predict disease severity.

**WHAT IS NEW**

✓ This study demonstrates that machine learning based systems may be used to guide fluid therapy with improved clinical results.
✓ Specifically machine learning guided fluid therapy may reduce the likelihood of over-resuscitation and subsequent adverse events including pulmonary insufficiency.

## Supplementary Material
