# Supplementary material for: Machine Learning–Guided Fluid Resuscitation for Acute Pancreatitis Improves Outcomes
Source: Clin Transl Gastroenterol. 2025 Jan 24;16(4):e00825. doi: 10.14309/ctg.0000000000000825 (PMC12020695; doi:10.14309/ctg.0000000000000825)
Supplement: Supplementary file 1 [file ct9-16-e00825-s001.docx]

­

n = 70 patients excluded for history of chronic pancreatitis

n = 896 patients after exclusion criteria applied

**Supplementary Figure 1** demonstrates sequential exclusion criteria for analysis based on ADAPT output. Initially n= 1409 patients were considered, with exclusion of n = 70 patients for chronic pancreatitis, n = 75 patients with history of US or CT findings of cirrhosis in the last 6 months, n = 85 patients excluded with history of stage IV or V CKD, n = 18 patients with NYHA III or IV heart failure, n = 24 patients for being transferred to outside hospital or left against medical advice, n = 54 patients for lacking data, and n = 187 patients for being under resuscitated (more than 500cc under ADAPT recommendation). Ultimately, n = 896 patients were evaluated with n = 700 falling under the over resuscitation group (>500 cc above ADAPT recommendation) and n = 196 falling under the adequately resuscitation group (within 500cc of ADAPT recommendation).

n= 196 (22%)

Adequately resuscitated group,
<=500cc within ADAPT recommendation.

n= 700 (78%)

Over resuscitated group,
>500cc over ADAPT recommendation.

n = 187 patients excluded for under resuscitated group, >500cc under ADAPT recommendation.

n = 24 patients excluded for being transferred to outside hospital or left against medical advice

n = 85 patients excluded for history of stage IV or V CKD

n = 54 patients excluded for lacking data

n = 75 patients excluded for CT or US showing cirrhosis within 6 months of admission

n = 18 patients excluded for NYHA III or IV heart failure

n = 1409 total patients with acute pancreatitis
