# Supplementary material for: Machine Learning–Guided Fluid Resuscitation for Acute Pancreatitis Improves Outcomes
Source: Clin Transl Gastroenterol. 2025 Jan 24;16(4):e00825. doi: 10.14309/ctg.0000000000000825 (PMC12020695; doi:10.14309/ctg.0000000000000825)
Supplement: Supplementary file 2 [file ct9-16-e00825-s002.docx]

**Supplementary Table 1: Clinical Outcomes of Over-Resuscitated versus Adequately-Resuscitated Adjusted by Cardinal Severity Scores and Charlson Comorbidity Index**

| **Outcome**  **Covariate** | | **Baseline SIRS** | **Panc3**  **Severe** | **Marshall’s MOF** | **HAPS Severe** | **Glasgow**  **OR** | **Glasgow Severe** | **BISAP** | **BISAP Severe** | **Charlson Comorbidity Index** |
| --- | --- | --- | --- | --- | --- | --- | --- | --- | --- | --- |
|  | | **Odds Ratio (95% Confidence Interval*)** | | | | | | | | |
| **New Respiratory Failure** | | **2.73(1.06-7.03)** | **2.87(1.12-7.34)** | **2.85(1.11-7.30)** | **2.77(1.08-7.12)** | **2.66(1.03-6.87)** | **2.78(1.08-7.11)** | 2.60(1.00-6.74) | **2.72(1.05-7.02)** | **2.89(1.11-7.47)** |
| **New Circulatory Failure** | | 0.97(0.20-4.64) | 1.10(0.23-5.22) | 1.13(0.24-5.42) | 1.19(0.25-5.69) | 0.98 (0.2-4.77) | 0.99(0.2-4.77) | 0.99(0.2-4.78) | 1.04(0.22-4.99) | 0.93(0.19-4.47) |
| **New Renal Failure** | | 0.51(0.13-2.05) | 0.53(0.13-2.14) | 0.52(0.13-2.10) | 0.53(0.13-2.11) | 0.45(0.11-1.85) | 0.46(0.11-1.91) | 0.49(0.12-2.01) | 0.54(0.14-2.17) | 0.51(0.13-2.08) |
| **Local Pancreatitis Complications** | | **2.93(1.23-6.96)** | **2.90(1.22-6.87)** | **2.98(1.26-7.05)** | **3.02(1.28-7.17)** | **2.85(1.20-6.77)** | **2.94(1.24-6.96)** | **2.84(1.20-6.74)** | **2.99(1.26-7.07)** | **2.89(1.21-6.88)** |
|  | **Pancreatic Pseudocyst** | 2.30(0.52-10.12) | 2.32(0.52-10.22) | 2.30(0.52-10.12) | 2.38(0.54-10.50) | 2.43(0.55-10.73) | 2.36(0.54-10.40) | 2.36(0.54-10.41) | 2.36(0.54-10.37) | 2.34(0.53-10.38) |
|  | **Necrosis** | 2.75(0.95-7.95) | 2.64(0.92-7.60) | 2.77(0.96-7.99) | 2.77(0.96-8.00) | 2.53(0.88-7.32) | 2.74(0.95-7.90) | 2.60(0.90-7.51) | 2.73(0.95-7.85) | 2.75(0.95-7.95) |
|  | **Organized Fluid Collection** | 2.11(0.62-7.19) | 2.17(0.64-7.37) | 2.14(0.63-7.29) | 2.36(0.69-8.10) | 2.04(0.60-6.96) | 2.10(0.61-7.16) | 2.05(0.60-7.01) | 2.17(0.64-7.40) | 2.11(0.62-7.22) |
| **New Pleural Effusion** | | 2.76(0.97-7.86) | 2.36(0.82-6.78) | 2.37(0.82-6.81) | 2.35(0.82-6.74) | 2.25(0.78-6.52) | 2.32(0.81-6.67) | 2.27(0.79-6.53) | 2.31(0.80-6.65) | 2.28(0.79-6.54) |
| **Moderately Severe Pancreatitis** | | 1.58(0.92-2.71) | 1.58(0.93-2.71) | 1.66(0.96-2.85) | 1.62(0.95-2.76) | 1.55(0.9-2.68) | 1.57(0.92-2.68) | 1.49(0.86-2.59) | 1.55(0.9-2.66) | 1.60(0.93-2.74) |
| **Severe Pancreatitis** | | 2.44(0.85-7.01) | 2.53(0.89-7.21) | 3.16(0.99-10.08) | 2.50(0.88-7.15) | 2.26(0.77-6.61) | 2.28(0.79-6.53) | 2.15(0.73-6.3) | 2.31(0.79-6.71) | 2.63(0.90-7.64) |
| **Required ICU Admission** | | **2.40(1.41-4.11)** | **2.38(1.41-4.01)** | **2.62(1.52-4.53)** | **2.50(1.47-4.27)** | **2.46(1.43-4.24)** | **2.41(1.43-4.07)** | **2.36(1.38-4.04)** | **2.37(1.39-4.02)** | **2.40(1.40-4.10)** |
| **ICU Length of Stay >48 Hours** | | 1.63(0.93-2.87) | **1.79(1.03-3.14)** | **1.94(1.09-3.44)** | **1.82(1.04-3.18)** | 1.77(1.00-3.13) | **1.83(1.04-3.19)** | 1.74(0.98-3.10) | 1.76(0.99-3.10) | **1.79(1.01-3.17)** |
| **Hospital Length of Stay >48** | | **1.87(1.19-2.94)** | **1.91(1.22-2.98)** | **1.94(1.24-3.03)** | **1.95(1.25-3.05)** | **1.90(1.22-2.98)** | **1.96(1.25-3.06)** | **1.89(1.21-2.96)** | **1.91(1.22-2.98)** | **1.87(1.20-2.93)** |
| **SIRS at 48 Hours after Admission** | | **1.73(1.08-2.77)** | **1.75(1.10-2.78)** | **1.80(1.13-2.87)** | **1.75(1.10-2.79)** | **1.73(1.08-2.79)** | **1.76(1.11-2.80)** | **1.72(1.07-2.75)** | **1.73(1.09-2.76)** | **1.74(1.09-2.77)** |
| **Death** | | 0.71(0.14-3.56) | 0.80(0.16-3.97) | 0.84(0.16-4.35) | 0.82(0.17-4.08) | 0.48(0.09-2.12) | 0.34(0.05-2.11) | 0.51(0.09-2.79) | 0.50(0.09-2.84) | 0.88(0.17-4.54) |
| **Readmission** | | 1.16(0.68-1.98) | 1.17(0.69-1.99) | 1.18(0.69-2.00) | 1.22(0.71-2.08) | 1.16(0.68-1.98) | 1.16(0.68-1.98) | 1.14(0.67-1.95) | 1.15(0.67-1.96) | 1.17(0.69-2.00) |
|  | | **Coefficient (95% Confidence Interval*)** | | | | | | | | |
| **Length of Stay** | | 1.08 (-0.33-2.48) | 1.23(-0.19-2.66) | 1.26(-0.16-2.68) | 1.26(-0.17-2.68) | 1.15(-0.25-2.55) | 1.03(-0.36-2.43) | 1.11(-0.30-2.53) | 1.21(-0.21-2.63) | 1.34(-0.09-2.76) |
| **BUN Max during Hospitalization** | | 1.10 (-0.53-2.72) | 1.12(-0.50-2.75) | 1.27(-0.31-2.85) | 1.16(-0.47-2.80) | 0.97(-0.55-2.49) | 0.76(-0.77-2.30) | 0.72(-0.76-2.21) | 0.87(-0.66-2.41) | 1.58(-0.01-3.16) |
| **First 24 Hour Total Oral Morphine Equivalent** | | **9.42(2.70-16.15)** | **9.58(2.85-16.30)** | **9.82(3.09-16.56)** | **9.31(2.62-16.00)** | **9.72(2.98-16.46)** | **10.01(3.27-16.75)** | **9.93(3.19-16.67)** | **10.11(3.39-16.83)** | **9.13(2.42-15.83)** |
| **First 48 Hour Total Oral Morphine Equivalent** | | 6.39 (-0.94-13.72) | 6.75(-0.60-14.10) | 6.92(-0.42-14.27) | 6.53(-0.81-13.87) | 6.85(-0.51-14.21) | 7.22(-0.14-14.59) | 6.93(-0.44-14.30) | 7.22(-0.13-14.58) | 6.70(-0.66-14.07) |

***All analyses are multivariate controlling for gender, pancreatitis etiology, and baseline SIRS**

**Supplementary Table 2: Clinical Outcomes for Patients with Elevated HAPS Score**

| **Outcomes** | | **Adequately Resuscitated**  **N (%)** | **Over Resuscitated**  **N (%)** | **Odds ratio** | **95% Conf. interval** |
| --- | --- | --- | --- | --- | --- |
| **HAPS Positive** | | 81 (41.3) | 349 (49.9) | 1.41 | **1.02, 1.95** |
| **Peritonitis on Admission** | | **0** | 3 (0.4) |  |  |
| **Cr ≥ 2 on Admission** | | **0** | 8 (1.1) |  |  |
| **Hct ≥ 43 on Admission** | | 81 (41.3) | 346 (49.4) | 1.39 | **1.01, 1.91** |
|  | | **Adequately Resuscitated**  **N (%)** | **Over Resuscitated**  **N (%)** | **Odds ratio*** | **95% Conf. interval** |
| **New Respiratory Failure** | | 1 (1.2) | 35 (10.0) | 9.91 | **1.32, 74.32** |
| **New Circulatory Failure** | | **0** | 3 (0.9) |  |  |
| **New Renal Failure** | | 2 (2.5) | 6 (1.7) | 1.35 | 0.15, 11.83 |
| **Local Pancreatitis Complications** | | 3 (3.7) | 53 | 4.80 | **1.12, 20.64** |
|  | **Pancreatic Pseudocyst** | **0** | 8 |  |  |
|  | **Necrosis** | 1 (1.2) | 33 | 6.78 | 0.89, 51.66 |
|  | **Organized Fluid Collection** | 2 (2.5) | 12 | 3.01 | 0.38, 2.88 |
| **New Pleural Effusion** | | 1 (1.2) | 24 (6.9) | 4.66 | 0.61, 35.84 |
| **Moderately Severe Pancreatitis** | | 8 (9.9) | 67 (19.2) | 3.32 | **1.27, 8.67** |
| **Severe Pancreatitis** | | **0** | 30 (8.6) |  |  |
| **Required ICU Admission** | | 10 (12.4) | 95 (27.2) | 2.72 | **1.27, 5.82** |
| **ICU Length of Stay >48 Hours** | | 6 (7.4) | 55 (15.8) | 2.49 | **1.08, 5.73** |
| **Hospital Length of Stay >48** | | 63 (77.8) | 286 (82.0) | 2.33 | **1.18, 4.62** |
| **SIRS at 48 Hours after Admission** | | 11 (13.6) | 97 (27.8) | 2.71 | **1.29, 5.67** |
| **Death** | | **0** | 4 (1.2) |  |  |
| **Readmission** | | 5 (6.2) | 37 (10.6) | 1.65 | 0.62, 4.41 |
|  | | **Adequately Resuscitated**  **Mean (SD)** | **Over Resuscitated**  **Mean (SD)** | **Coefficient*** | **95% Conf. interval** |
| **Length of Stay** | | 5 (4) | 7 (12) | 2.52 | -0.22, 5.26 |
| **BUN Max during Hospitalization** | | 13 (5) | 17 (13) | 2.96 | -0.01, 5.94 |
| **First 24 Hour Total Oral Morphine Equivalent** | | 28.5 (33.7) | 43.4 (48.4) | **14.23** | **2.55, 25.92** |
| **First 48 Hour Total Oral Morphine Equivalent** | | 13.3 (23.2) | 32.4 (56.4) | **18.55** | **5.31, 31.79** |

***All analyses are multivariate controlling for gender, pancreatitis etiology, and baseline SIRS**

**Supplementary Table 3: Baseline Features of Under-Resuscitated**

| **Table 1 Baseline Features of Population** | | **Adequately Resuscitated** | | **Under Resuscitated** | | **p-Value** |
| --- | --- | --- | --- | --- | --- | --- |
|  |  | **Mean** | **SD** | **Mean** | **SD** |  |
| **ADAPT Recommended Fluid First 24 hours (mL)** | | 2435.0 | 417.0 | 2540.7 | 484.6 | **0.02** |
| **Actual Fluid Administered First 24 hours (mL)** | | 2465.4 | 539.3 | 1115.9 | 762.4 | **0.00** |
| **Mean Difference ADAPT versus Actual Fluid** | | 30.3 | 294.1 | -1422.7 | 720.5 | **0.00** |
| **Age (years)** | | 45 | 16 | 45 | 16 | 0.91 |
| **BMI (kg/m2)** | | 32.7 | 12.8 | 30.3 | 9.93 | 0.23 |
| **BUN (mg/dL)** | | 13.4 | 6.3 | 14.4 | 9.0 | 0.46 |
| **Creatinine (mg/dL)** | | 0.75 | 0.22 | 0.82 | 0.33 | **0.01** |
| **GFR (ml/min)** | | 105 | 23 | 103 | 26 | 0.62 |
| **Calcium (mg/dL)** | | 9.2 | 0.8 | 9.2 | 0.7 | 0.70 |
| **Lipase (U/L)** | | 2076 | 2081 | 1802 | 2069 | 0.20 |
| **Total Cholesterol (mg/dL)** | | 221 | 182 | 196 | 106 | 0.41 |
| **Pain Score (visual analogue)** | | 7.4 | 3.1 | 7.1 | 3.0 | 0.51 |
|  | | **N** | **%** | **N** | **%** | **p-Value** |
| **Male** | | 89 | 45.4 | 111 | 59.4 | **0.01** |
| **Female** | | 107 | 54.6 | 76 | 40.6 |  |
| **Race** | **Hispanic** | 169 | 86.2 | 147 | 78.6 | 0.33 |
|  | **White** | 6 | 3.1 | 11 | 5.9 |  |
|  | **Black** | 8 | 4.1 | 12 | 6.4 |  |
|  | **Asian** | 6 | 3.1 | 10 | 5.4 |  |
|  | **Other** | 7 | 3.6 | 7 | 3.7 |  |
| **Current Smoker** | | 27 | 13.9 | 27 | 14.8 | 0.11 |
| **Heavy EtOH** | | 36 | 18.5 | 36 | 19.8 | 0.32 |
| **FHx of Pancreatic Cancer** | | 1 | 0.5 | 0 | 0.0 | 0.37 |
| **Etiology** | **EtOH** | 26 | 13.8 | 30 | 16.8 | 0.32 |
|  | **Gallstone** | 112 | 59.3 | 91 | 50.8 |  |
|  | **Other** | 51 | 27.0 | 57 | 31.8 |  |
| **SIRS at Admission** | | 38 | 19.4 | 20 | 10.7 | **0.02** |
| **DM** | **Type 1** | 6 | 3.1 | 6 | 3.2 | 0.75 |
|  | **Type 2** | 45 | 23.0 | 37 | 19.8 |  |
| **Insulin** | | 15 | 8.0 | 15 | 8.7 | 0.81 |
| **HTN** | | 45 | 23.3 | 44 | 24.2 | 0.33 |
| **HLD** | | 23 | 11.9 | 24 | 13.3 | 0.68 |
| **Statin** | | 16 | 8.3 | 24 | 13.6 | 0.11 |
| **Fibrate** | | 5 | 2.6 | 2 | 1.1 | 0.30 |
| **CKD** | **1** | 149 | 76.0 | 142 | 75.9 | 0.08 |
|  | **2** | 39 | 19.9 | 28 | 15.0 |  |
|  | **3** | 8 | 4.1 | 17 | 9.1 |  |

**Supplementary Table 4: Clinical Outcomes of Under-Resuscitated versus Adequately-Resuscitated**

| **Outcomes** | | **Adequately Resuscitated**  **N (%)** | **Under Resuscitated**  **N (%)** | **Odds ratio*** | **95% Conf. interval** |
| --- | --- | --- | --- | --- | --- |
| **New Respiratory Failure** | | 2 (1.0) | 4 (2.1) | 1.04 | 0.26, 4.22 |
| **New Circulatory Failure** | | 2 (1.0) | 1 (0.5) | 0.50 | 0.04, 6.27 |
| **New Renal Failure** | | 5 (2.6) | 3 (1.6) | 0.78 | 0.12, 4.98 |
| **Local Pancreatitis Complications** | | 9 (5.8) | 5 (2.7) | 0.83 | 0.24, 2.89 |
|  | **Pancreatic Pseudocyst** | 2 (1.3) | 3 (1.6) | 1.68 | 0.27, 10.59 |
|  | **Necrosis** | 4 (2.6) | 5 (2.7) | 1.04 | 0.25, 4.40 |
|  | **Organized Fluid Collection** | 3 (1.9) | 4 (2.1) | 1.56 | 0.33, 7.45 |
| **New Pleural Effusion** | | 4 (2.0) | 3 (1.6) | 0.97 | 0.21, 4.58 |
| **Moderately Severe Pancreatitis** | | 17 (8.7) | 16 (8.6) | 0.88 | 0.42, 1.86 |
| **Severe Pancreatitis** | | 4 (2.0) | 5 (2.7) | 1.68 | 0.43, 6.65 |
| **Required ICU Admission** | | 19 (9.7) | 16 (8.6) | 0.93 | 0.43, 2.02 |
| **ICU Length of Stay >48 Hours** | | 12 (6.1) | 10 (5.3) | 0.57 | 0.23, 1.40 |
| **Hospital Length of Stay >48** | | 156 (79.6) | 139 (74.3) | 0.82 | 0.49, 1.38 |
| **SIRS at 48 Hours after Admission** | | 27 (13.8) | 15 (8.0) | 0.44 | 0.20, 0.98 |
| **Death** | | 2 (1.0) | 2 (1.1) | 1.14 | 0.15, 8.81 |
| **Readmission** | | 20 (10.2) | 28 (15.0) | 1.70 | 0.87, 3.29 |
|  | | **Adequately Resuscitated**  **Mean (SD)** | **Under Resuscitated**  **Mean (SD)** | **Coefficient*** | **95% Conf. interval** |
| **Length of Stay** | | 6 (4) | 5 (5) | 0.19 | -0.72, 1.10 |
| **BUN Max during Hospitalization** | | 14 (7) | 15 (10) | 1.56 | -0.26, 3.38 |
| **First 24 Hour Total Oral Morphine Equivalent** | | 27.3 (35.8) | 20.2 (15.1) | -7.43 | -14.54, -0.31 |
| **First 48 Hour Total Oral Morphine Equivalent** | | 19.4 (37.5) | 15.1 (31.5) | -4.02 | -11.47, 3.43 |

***All analyses are multivariate controlling for gender, pancreatitis etiology, and baseline SIRS**
